# Supplementary material for: Multinucleation associated DNA damage blocks proliferation in p53-compromised cells
Source: Commun Biol. 2021 Apr 9;4:451. doi: 10.1038/s42003-021-01979-5 (PMC8035210; doi:10.1038/s42003-021-01979-5)
Supplement: Supplementary file 1 — Supplementary Information [file 42003_2021_1979_MOESM1_ESM.pdf]

Multinucleation Associated DNA Damage blocks proliferation in p53-compromised cells

Madeleine Hart, Sophie D Adams and Viji M Draviam*

Supplementary Materials

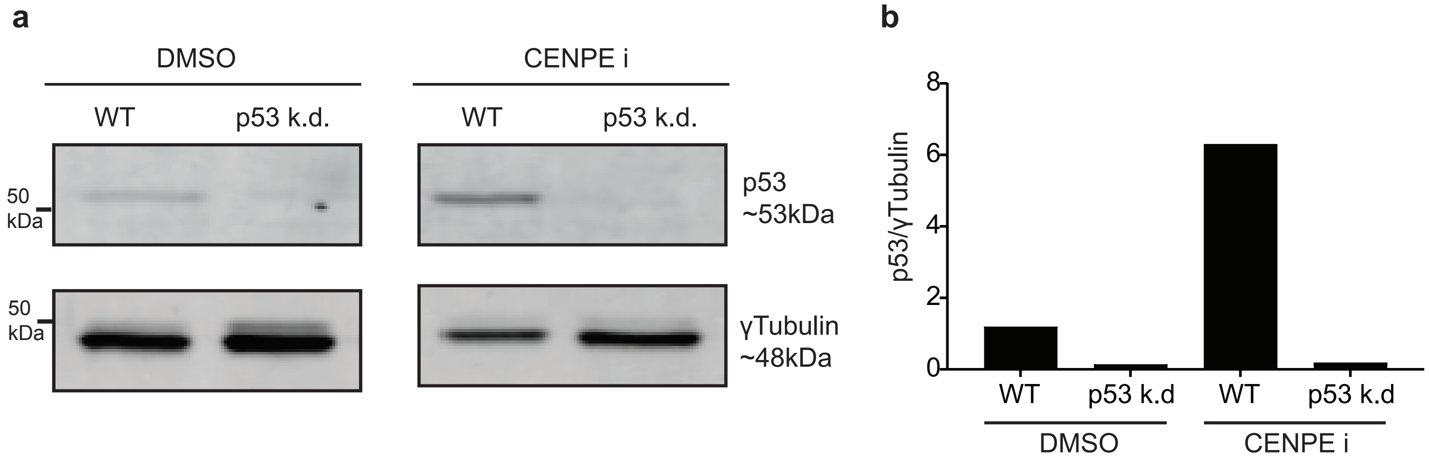

Supplementary Fig. 1: Extent of p53 knockdown (detailed legend page-8 onwards).

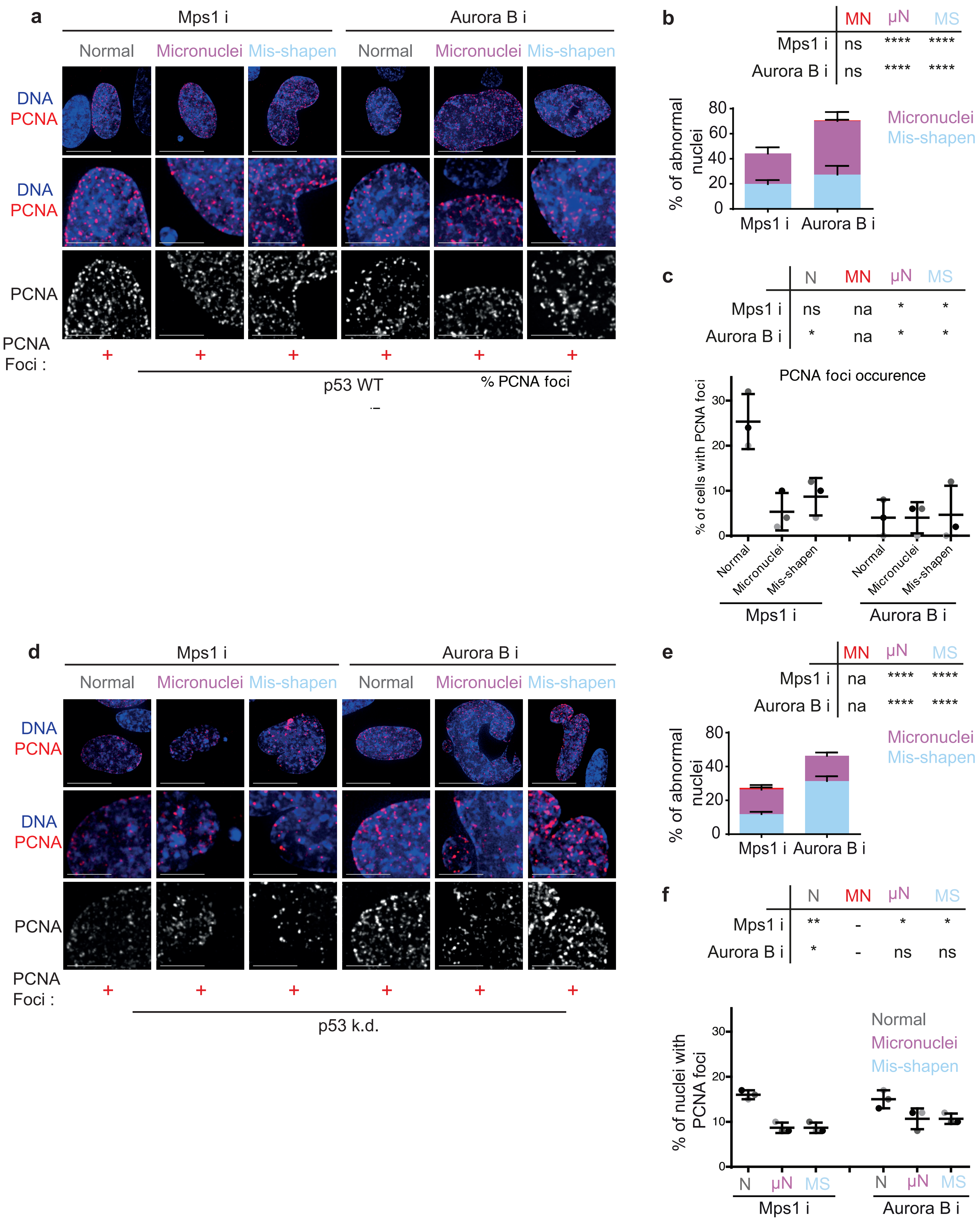

Supplementary Fig. 2: Mis-shapen and micronucleated cells have similar cell cycle responses following an accelerated mitotic progression (detailed legend page-8 onwards).

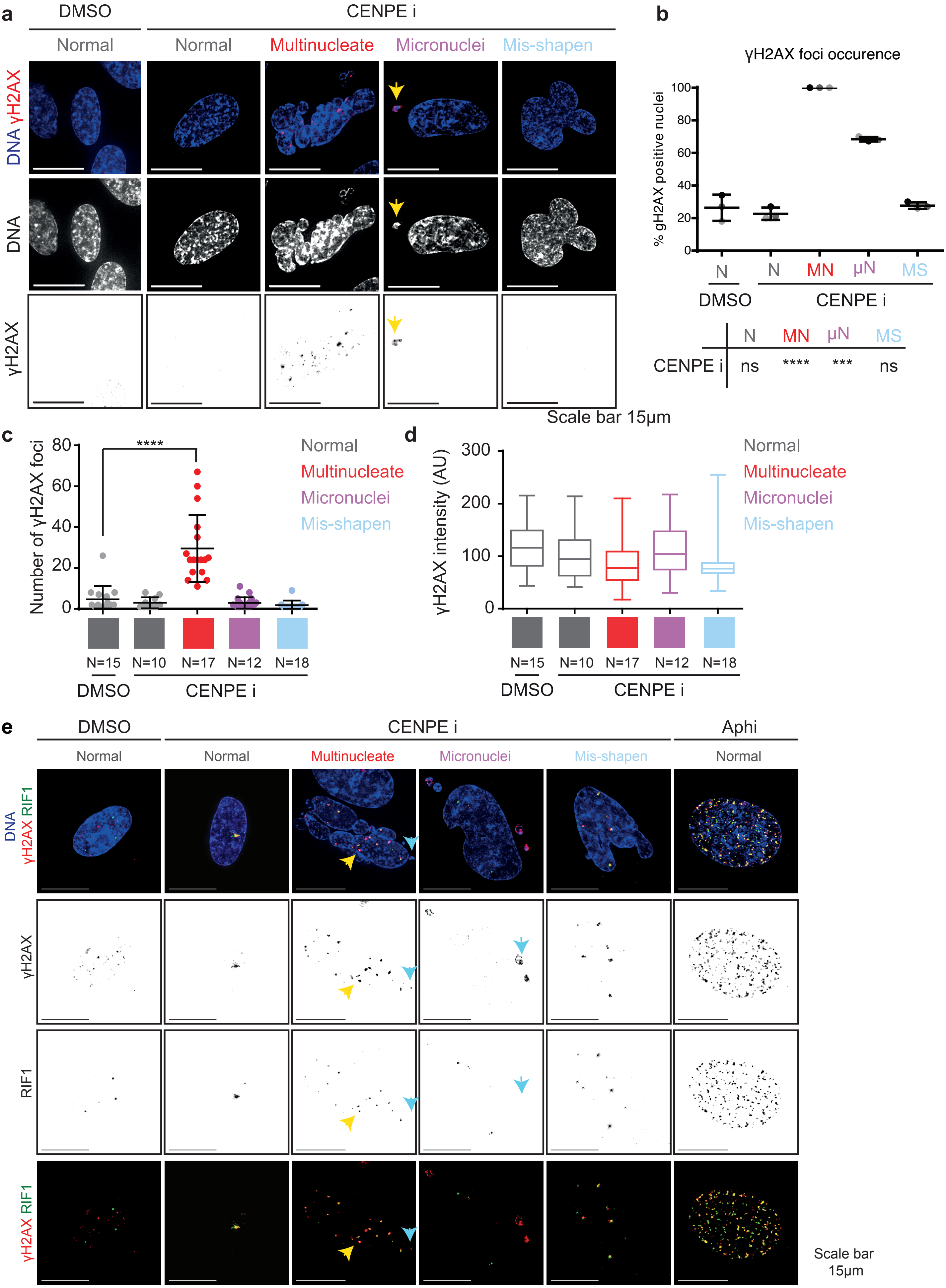

Supplementary Fig. 3: Multinucleation induces DNA damage and gamma H2AX foci

(detailed legend page-8 onwards).

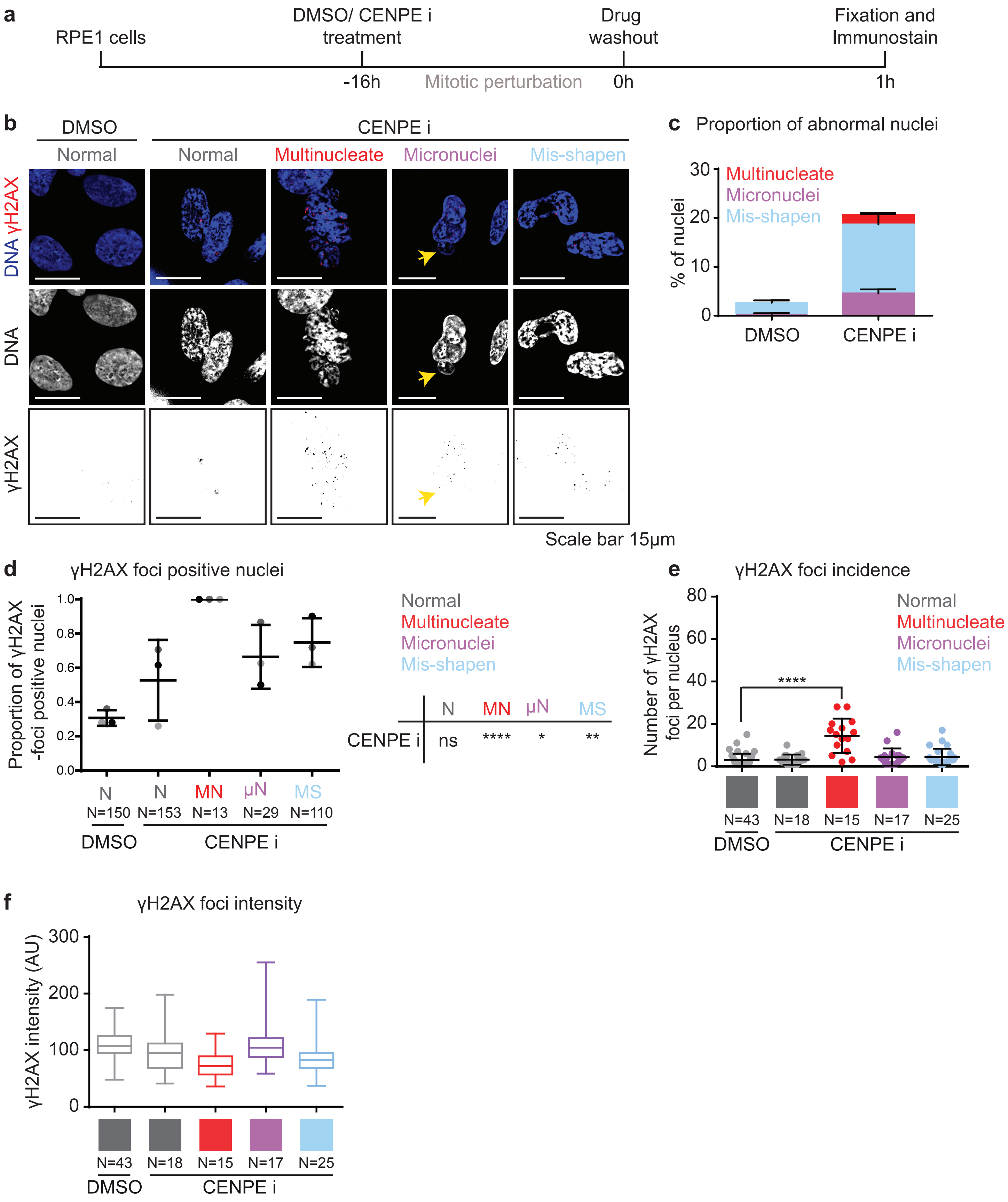

Supplementary Fig. 4: Multinucleate cells have DNA damage 1-hour post-drug washout
(detailed legend page-8 onwards).

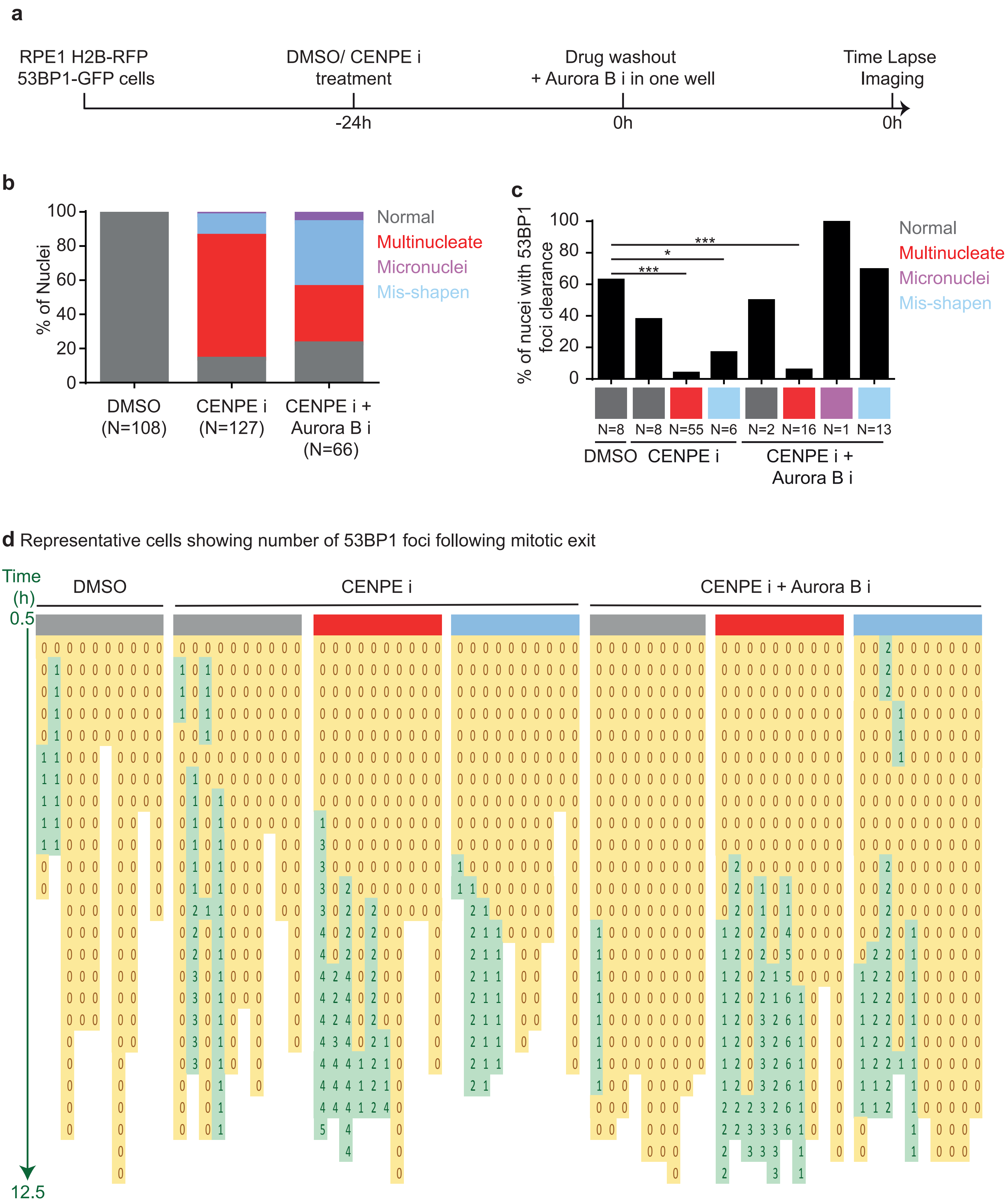

Supplementary Fig. 5: Individual tracking of 53BP1-GFP foci dynamics
(detailed legend page-8 onwards).

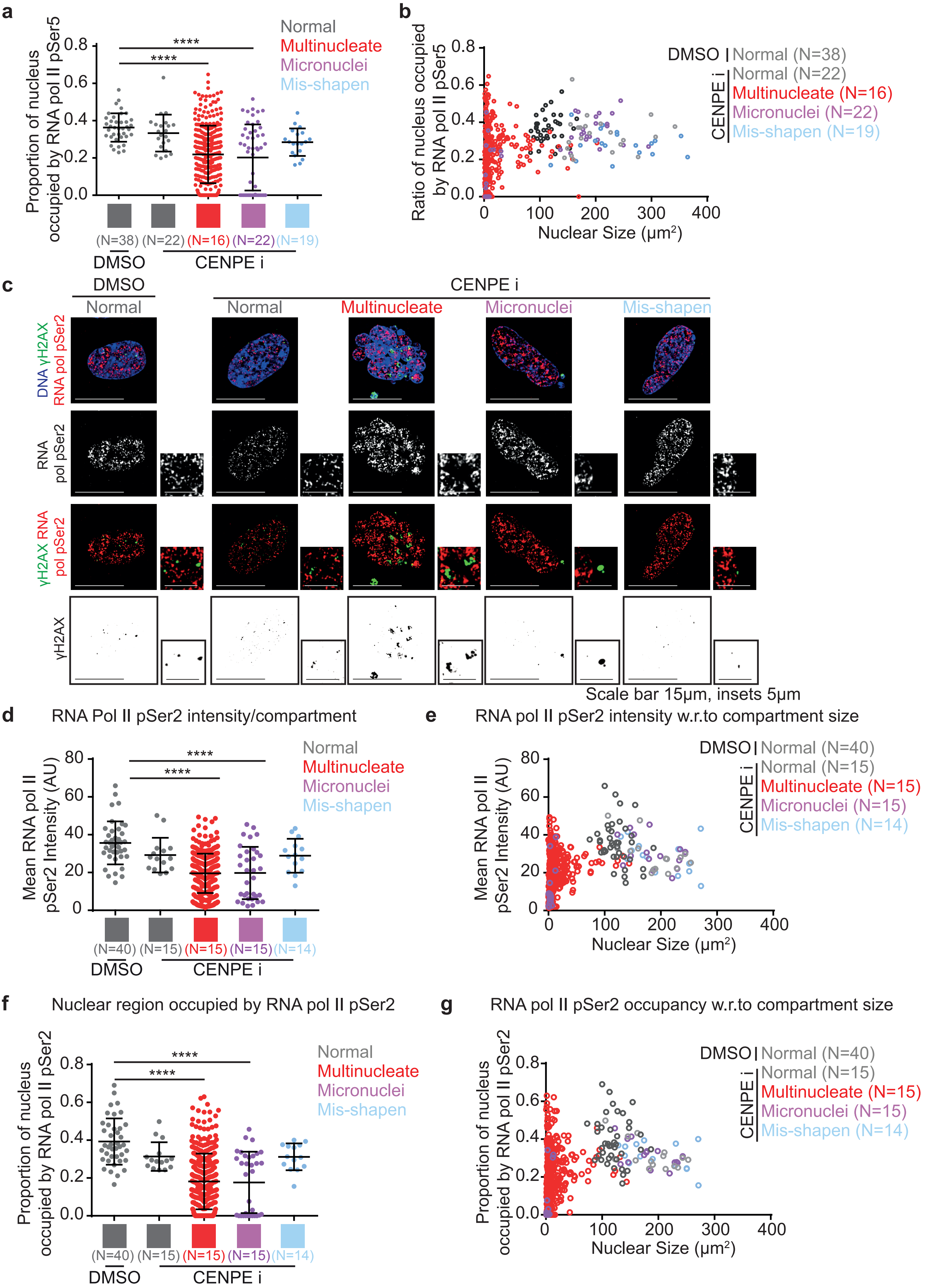

Supplementary Fig. 6: Transcription initiation and elongation variable in smaller compartments of multinucleate cells (detailed legend page-8 onwards).

a Immunoblot corresponding to Supplementary Fig.1a

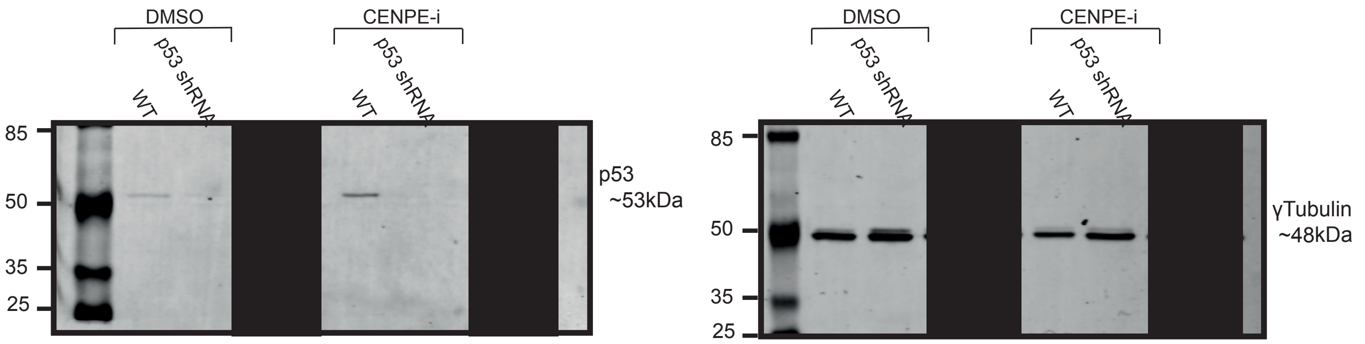

b Immunoblot corresponding to Fig. 5a

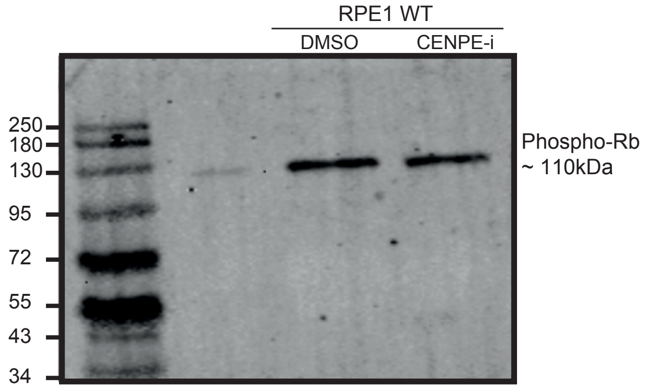

c Immunoblot corresponding to Fig. 5d

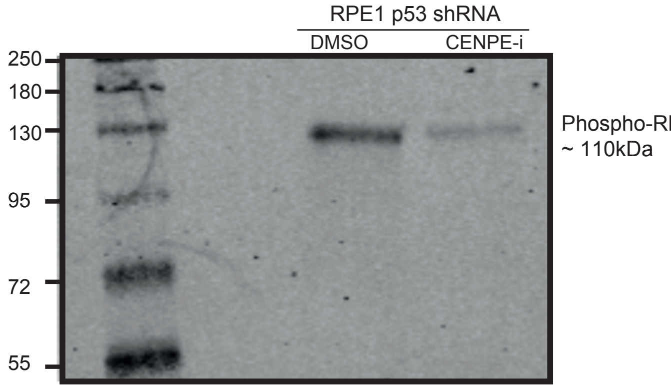

Supplementary Fig. 7: Larger immunoblots of cropped images
(detailed legend page-8 onwards).

Multinucleation Associated DNA Damage blocks proliferation in p53-compromised cells

Madeleine Hart, Sophie D Adams and Viji M Draviam*

Supplementary Materials

Supplementary Fig. 1: Extent of p53 knockdown.
RPE1 p53 WT and RPE1 H2B-GFP p53 k.d. cells were treated with either DMSO (solvent control) or CENPE inhibitor for 16 hours. Drug treatments were then washed out and cells were lysed 2 days later. **a** Lysates were separated using SDS-PAGE and immunoblotted with antibodies against p53 and gamma-tubulin. **b** Quantification of immunostained bands; p53 band intensities were normalised to the intensity of gamma-tubulin bands.

Supplementary Fig. 2: Mis-shapen and micronucleated cells have similar cell cycle responses following an accelerated mitotic progression.
RPE1 p53 WT or RPE1 H2B-GFP p53 k.d. cells were treated with MPS1 inhibitor or Aurora B inhibitor for 16 hours then washed out. 48 hours later cells were fixed and immunostained with an antibody against PCNA and DNA was stained with DAPI. **a** Representative images of RPE1 p53 WT cells treated with MPS1 or Aurora B inhibitor. Scale bar 15µm, insets 5µm. **b** Quantification of nuclear morphology changes following indicated treatments in RPE1 p53 WT cells. N=600 cells from across 3 independent repeats. Statistical analysis was carried out using a two-way ANOVA with multiple comparisons and a confidence interval of 95%. **c** Quantification of the percentage of PCNA-foci positive cells, within each nuclear morphology bin, following MPS1 or Aurora B inhibition. N= at least 150 nuclei from across 3 independent repeats shown as shades of grey. Statistical analysis was carried out using multiple unpaired t-tests, comparing each morphology after MPS1 or Aurora B

inhibition to normal nuclei after DMSO treatment. **d** Representative images of RPE1 H2B-GFP p53 k.d. cells treated with MPS1 or Aurora B inhibitor. Scale bar 15μm, insets 5μm. **e** Quantification of nuclear morphology changes following MPS1 or Aurora B inhibitor treatment in RPE1 H2B-GFP p53 k.d. cells. N=900 cells from across 3 independent repeats. Statistical analysis was carried out using a two-way ANOVA with multiple comparisons and a confidence interval of 95%. **f** Quantification of the percentage of PCNA-foci positive cells, within each nuclear morphology bin, following MPS1 or Aurora B inhibition. N= at least 150 nuclei from across 3 independent repeats shown as shades of grey. Statistical analysis was carried out using multiple unpaired t-tests, comparing each morphology after MPS1 or Aurora B inhibition to normal nuclei after DMSO treatment.

Supplementary Fig. 3: Multinucleation induces DNA damage and gamma H2AX foci

RPE1 cells were treated with either DMSO or CENPE inhibitor for 16 hours, then washed out and fixed 2 days later. Cells were immunostained with antibodies against γH2AX and DNA was stained with DAPI. **a** Representative images of each nuclear morphology after CENPE inhibition or DMSO treatment. Images are maximum z projections of at least 4 images spanning 2μm. Scale bar 15μm. Yellow arrow indicates a micronucleus. **b** Graph to show the percentage of each nuclear morphology which had γH2AX foci within the nucleus, following DMSO or CENPE inhibition. N=300 nuclei per morphology from across 3 independent repeats shown as shades of grey. Significance was assessed with multiple unpaired t-tests to compare each nuclear morphology after CENPE inhibition to normal nuclei after DMSO treatment. **c** Quantification of the number of γH2AX foci per nucleus, in nuclei with γH2AX foci. Quantification is divided based upon nuclear morphology after DMSO or CENPE inhibition. N indicated the number of cells, from across 3 independent repeats. Significance was assessed using an unpaired t-test. Other nuclear morphologies were non-significant when compared to normal nuclei after DMSO treatment. **d** Quantification of the γH2AX fluorescence intensity per foci, in nuclear morphologies indicated after DMSO or CENPE inhibitor treatments. Boxes represent second and third quartiles respectively, middle line displays median, and whiskers span minimum to maximum values. N indicated the number of cells analysed, taken across 3 independent repeats. **e** RIF1 localises to sites of DNA

damage in multinucleate cells, but not inside micronuclei. RPE1 cells were treated with DMSO or CENPE inhibitor for 16 hours then washed out and 2 days later fixed and immunostained with antibodies against gamma H2AX and RIF1. DNA was stained with DAPI. As a positive control RPE1 cells were treated with Aphidicolin for the duration of the experiment. Representative images of cells with differing nuclear morphology after indicated drug treatments; Normal after DMSO (N=29 cells), Normal (N=8), Multinucleate (N=12), Micronuclei (N=8) and Mis-shapen nuclei (N=7) after CENPE inhibition, and normal nuclei after Aphidicolin treatment (N=8). Blue arrow indicates gamma H2AX foci but no RIF1 localisation within micronucleus, and yellow arrow indicates site of gamma H2AX and RIF1 co-localisation. Scale bar 15μm.

Supplementary Fig. 4: Multinucleate cells have DNA damage 1-hour post-drug washout.

a Experimental regime; RPE1 cells were treated with CENPE inhibitor or DMSO for 16 hours, the drug then washed out and cells were fixed 1 hour later. Cells were immunostained with antibodies against γH2AX and DNA was stained with DAPI. **b** Representative images of nuclei as treated as indicated in A. Scale bar 15μm. Yellow arrow indicates a micronucleus. **c** Quantification of the proportion of cells with abnormal nuclei after treatment as described in A. N=900 cells per condition, taken from 3 independent repeats. **d & e** Quantification of either the proportion of each nuclear morphology positive for γH2AX foci (d) or the number of γH2AX foci in each nuclear morphology (e). N is indicated below bars, taken from 3 independent repeats. Statistical analysis was carried out using multiple unpaired t-tests, comparing each morphology after CENPE inhibition to normal nuclei after DMSO treatment. **f** Quantification of the number of γH2AX foci per nucleus, for each nuclear morphology, after DMSO or CENPE inhibitor treatment. N is indicated under each bar, taken from 3 independent repeats. Significance was assessed using an unpaired t-test. Other nuclear morphologies were non-significant when compared to normal nuclei after DMSO treatment. **g** Quantification of the fluorescence intensity of γH2AX, per foci, in nuclear morphologies indicated after DMSO or CENPE inhibitor treatment. Boxes represent second and third quartiles respectively, middle line

displays median and whiskers span minimum to maximum values. N indicated the number of cells analysed, taken across 3 independent repeats.

Supplementary Fig. 5: Individual tracking of 53BP1-GFP foci dynamics.

a Experimental regime; RPE1 H2B-RFP 53BP1-GFP cells were treated with DMSO or CENPE inhibitor for 24 hours, then washed out and one well additionally treated with Aurora B inhibitor. Cells were then imaged for at least 12 hours with an image every 30 minutes. **b** Quantification of the nuclear morphology of daughter cells exiting mitosis after DMSO, CENPE inhibitor or CENPE inhibitor plus Aurora B inhibitor treatment. N, as indicated, is the number of daughter nuclei. **c** Quantification of the proportion of nuclei which display clearance of 53BP1 foci during time-lapse imaging. N values below bars indicate the number of nuclei. Statistical significance was assessed using a proportions test, with a 95% confidence interval. *** indicates $p<0.001$, * indicates $p<0.05$. **d** Representative tracks of individual cells with the number of 53BP1 foci over time following mitotic exit. The number and colour represent the number of 53BP1 foci per nucleus.

Supplementary Fig. 6: Transcription initiation and elongation variable in smaller compartments of multinucleate cells.

a Quantification of the proportion of the nucleus occupied by RNA pol II CTD pSer5, for each nucleus (normal and misshapen nuclei) or nuclear compartment (micronuclei and multinucleate). N values indicate the number of cells analysed. Statistical significance was assessed using a one way ANOVA with multiple comparisons. **b** Plot of the proportion of the nucleus occupied by RNA pol II CTD pSer5 against nucleus or nuclear compartment size. Colours of plots represent nuclear morphology as indicated. N values indicate the number of cells. **c - g** RPE1 cells were treated with DMSO (control) or CENPE inhibitor for 16 hours, then washed out and fixed 2 days later. Cells were immunostained with antibodies against γH2AX and RNA polymerase II CTD pSer2 and DNA was stained with DAPI. **c** Representative images of differing nuclear morphologies after CENPE inhibitor treatment, and normal nuclei after DMSO treatment. Scale bar 15μm, insets 5μm. **d** Quantification of the average RNA pol II CTD pSer2 intensity per nucleus (normal or misshapen nuclei) or nuclear compartment (micronucleated and multinucleate). Each plot represents one nucleus/nuclear compartment respectively. N indicated below

the bar is the number of cells analysed, from across 3 independent repeats. Statistical significance was assessed using a one-way ANOVA with multiple comparisons. **e** Average RNA pol II CTD pSer2 intensity per nucleus/nuclear compartments plotted against the size of the nucleus/nuclear compartment. Colours of plots represent nuclear morphology as indicated. N, as indicated, is the number of cells taken from across 3 independent repeats. **f** Quantification of the proportion of the DNA region occupied by RNA pol II CTD pSer2, for each nucleus (normal and misshapen nuclei) or nuclear compartment (micronuclei and multinucleate). N values indicate the number of cells analysed. Statistical significance was assessed using a one way ANOVA with multiple comparisons. **g** Graph of the proportion of the nucleus occupied by RNA pol II CTD pSer5 against nucleus or nuclear compartment size. Colours of plots represent nuclear morphology as indicated.

Supplementary Fig. 7: Larger immunoblots of cropped images.

a-c Images of immunoblots corresponding to cropped images presented in Supplementary Fig. 1a (a), Fig. 5a (b) and Fig. 5d (c). Black boxes are placed in lanes irrelevant to the current study.
