# Supplementary material for: Multinucleation associated DNA damage blocks proliferation in p53-compromised cells
Source: Commun Biol. 2021 Apr 9;4:451. doi: 10.1038/s42003-021-01979-5 (PMC8035210; doi:10.1038/s42003-021-01979-5)
Supplement: Supplementary file 2 — Description of Additional Supplementary Files [file 42003_2021_1979_MOESM2_ESM.pdf]

Description of Additional Supplementary Files

File name: Supplementary Videos 1A, 1B, 1C

Description: Multinucleate cells gain 53BP1-GFP foci following mitotic exit. RPE1 H2B-RFP 53BP1-GFP cells were treated with DMSO or CENPE for 24 hours. Cells were then washed and one well was additionally treated with Aurora B inhibitor. Cells were then imaged for at least 12 hours using time-lapse microscopy. Imaging shows 53BP1-GFP (top left), H2B-RFP (top right), widefield (lower left) and merge (lower right) channels. a Normal daughter nuclei exiting mitosis following DMSO treatment, do not gain 53BP1-GFP foci during the course of the imaging. b A CENPE inhibitor arrested cell exits mitosis into a multinucleate daughter cell which gains initial 53BP1-GFP foci at 08:30 hrs and increases in number until the end of the video recording. Corresponding images in Fig. 5 (first two panels) present intensity adjustments to rule out any low intensity foci in early G1. c CENPE inhibitor arrested mitotic cell is released from mitosis with Aurora B inhibition and forms a multinucleate daughter cell. Numerous 53BP1-GFP foci appear at 09:00hrs which increase in number until the end of imaging.

File name: Supplementary Videos 2A, 2B, 2C

Description: Nuclear PCNA foci in normal nuclei after DMSO or CENPE inhibition but not in multinucleate cells, which have increasing p21 levels. RPE1 mRuby-PCNA p21-GFP cells were treated with DMSO (a) or CENPE inhibitor (b, c) for 16 hours. Drugs were then washed out and live-cell imaging started 10 hours later. Imaging shows p21-GFP (top left), mRuby-PCNA (top right), widefield (lower left) and merge (lower right) channels. a Normal nucleus after DMSO treatment shows nuclear PCNA foci and lack of p21. b Normal nucleus after CENPE inhibition shows nuclear PCNA foci and lack of p21. c Multinucleate cell after CENPE inhibition exits mitosis and builds p21 level through time, without gaining nuclear PCNA foci. Scale bar 25 μm. Videos correspond to images shown in Fig. 2.
