# Supplementary material for: Multinucleation associated DNA damage blocks proliferation in p53-compromised cells
Source: Commun Biol. 2021 Apr 9;4:451. doi: 10.1038/s42003-021-01979-5 (PMC8035210; doi:10.1038/s42003-021-01979-5)
Supplement: Supplementary file 9 — Reporting Summary [file 42003_2021_1979_MOESM9_ESM.pdf]

nature research

Corresponding author(s): Viji Draviam

Last updated by author(s): 11 March 2021

Reporting Summary

Nature Research wishes to improve the reproducibility of the work that we publish. This form provides structure for consistency and transparency in reporting. For further information on Nature Research policies, see our Editorial Policies and the Editorial Policy Checklist.

Statistics

For all statistical analyses, confirm that the following items are present in the figure legend, table legend, main text, or Methods section.

n/a	Confirmed	
☐	☒	The exact sample size (*n*) for each experimental group/condition, given as a discrete number and unit of measurement
☐	☒	A statement on whether measurements were taken from distinct samples or whether the same sample was measured repeatedly
☐	☒	The statistical test(s) used AND whether they are one- or two-sided<br>*Only common tests should be described solely by name; describe more complex techniques in the Methods section.*
☒	☐	A description of all covariates tested
☒	☐	A description of any assumptions or corrections, such as tests of normality and adjustment for multiple comparisons
☒	☐	A full description of the statistical parameters including central tendency (e.g. means) or other basic estimates (e.g. regression coefficient) AND variation (e.g. standard deviation) or associated estimates of uncertainty (e.g. confidence intervals)
☒	☐	For null hypothesis testing, the test statistic (e.g. *F*, *t*, *r*) with confidence intervals, effect sizes, degrees of freedom and *P* value noted<br>*Give P values as exact values whenever suitable.*
☒	☐	For Bayesian analysis, information on the choice of priors and Markov chain Monte Carlo settings
☒	☐	For hierarchical and complex designs, identification of the appropriate level for tests and full reporting of outcomes
☒	☐	Estimates of effect sizes (e.g. Cohen's *d*, Pearson's *r*), indicating how they were calculated

Our web collection on statistics for biologists contains articles on many of the points above.

Software and code

Policy information about availability of computer code

Data collection	SoftWorx for the collection of microscopy images
Data analysis	ImageJ for microscopy image analysis

For manuscripts utilizing custom algorithms or software that are central to the research but not yet described in published literature, software must be made available to editors and reviewers. We strongly encourage code deposition in a community repository (e.g. GitHub). See the Nature Research guidelines for submitting code & software for further information.

Data

Policy information about availability of data

All manuscripts must include a data availability statement. This statement should provide the following information, where applicable:

- Accession codes, unique identifiers, or web links for publicly available datasets
- A list of figures that have associated raw data
- A description of any restrictions on data availability

All numerical data associated with the paper have been uploaded to figshare and doi included in the manuscript.

Field-specific reporting

Life sciences study design

All studies must disclose on these points even when the disclosure is negative.

Sample size	Sample size was not statistically predetermined.
Data exclusions	No data was excluded. Only experimental repeats which were unsuccessful (increased cell death or change in protocol were disregarded).
Replication	Each experiment was repeated at least three times and where appropriate error bars representing standard deviation are shown.
Randomization	Cells were seeded using the same protocol and assigned to a drug regime, in separate wells/dishes. Controls were provided in the form of DMSO (solvent only) treated cells, which were treated in every other way (except drug treatment) the same. Additional control cells are those with normal nuclei on the same coverslip/dish as the nuclear abnormalities. These cells have undergone exactly the same treatment.
Blinding	Not applicable

Reporting for specific materials, systems and methods

We require information from authors about some types of materials, experimental systems and methods used in many studies. Here, indicate whether each material, system or method listed is relevant to your study. If you are not sure if a list item applies to your research, read the appropriate section before selecting a response.

Materials & experimental systems

n/a	Involved in the study
☐	☒ Antibodies
☐	☒ Eukaryotic cell lines
☒	☐ Palaeontology and archaeology
☒	☐ Animals and other organisms
☒	☐ Human research participants
☒	☐ Clinical data
☒	☐ Dual use research of concern

Methods

n/a	Involved in the study
☒	☐ ChIP-seq
☒	☐ Flow cytometry
☒	☐ MRI-based neuroimaging

Antibodies

Antibodies used	Anti p-53, Santa Cruz (sc-126), Anti Phospho-Rb (ser 807/811), CST (8516S), Anti- PCNA, CST (2586S), Anti gammaH2AX, Abcam (ab26350), Anti- RNA polymerase II CTD repeat phospho-Ser2, Abcam (ab126353), Anti RNA polymerase II CTD repeat phospho -Ser5, Abcam (Ab5408)
Validation	All antibodies were validated by manufacturers using molecular weight markers in western blots.

Eukaryotic cell lines

Policy information about cell lines

Cell line source(s)	ATCC (RPE1 parental cell line) and research groups that generated the fluorescent reporter cell lines
Authentication	none of the cell lines used were authenticated, but the authors primarily grow RPE1 cell lines.
Mycoplasma contamination	RPE1 parental cell line was tested for Mycoplasma contamination 5 years ago.
Commonly misidentified lines (See ICLAC register)	not applicable
